# Budd–Chiari Syndrome Management: Controversies and Open Issues

**DOI:** 10.3390/diagnostics12112670

**Published:** 2022-11-03

**Authors:** Andrea Mancuso

**Affiliations:** Centro di Riferimento Regionale Malattie Rare, Sindrome di Budd–Chiari e Teleangectasia Emorragica Ereditaria, Medicina Interna 1, ARNAS Civico-Di Cristina-Benfratelli, Piazzale Leotta 4, 90100 Palermo, Italy; mancandrea@libero.it; Tel.: +39-32-9899-7893; Fax: +39-09-1609-0252

## 1. Diagnostic Controversies in Budd–Chiari Syndrome

Budd–Chiari Syndrome (BCS) is due to thrombosis of hepatic veins (HVs), inferior vena cava (IVC) or both, leading to impaired hepatic venous outflow. Usually, one or more prothrombotic conditions, such as myeloproliferative disorders (MPD), are found. However, a number of cases remain idiopathic [1,2,3,4,5]. Furthermore, prothrombotic disorders are more frequent in the West than in Asia, where HVs involvement is more common and BCS is generally due to IVC involvement. Additionally, prothrombotic factors such as MPD are less frequent. However, despite similar physiopathology, management may be different in the West and the East [6]. It seems realistic to speculate that BCS in the West and East is the same syndrome within different diseases [7]. Recently, however, new perspectives of BCS have begun to emerge [1].

Doppler ultrasound (US) is the main imaging method used to investigate liver dysfunction upon presentation. Historically, Doppler US for BCS diagnosis—the criteria of which is probably in need of refinement—standardized specific signs (HV non-visualization, fibrous cord, thrombosis, and stenosis), suggestive signs (evidence of intrahepatic collateral circulation, caudate vein 3 mm or larger), and signs shared with other conditions (regenerative nodules, caudate lobe hypertrophia, nonhomogeneous parenchymal structure, portal thrombosis, re-canalized umbilical vein, ascites, etc.) [8]. However, in skilled hands, evidence of intrahepatic collateral circulation is the most frequent sign at Doppler US. Furthermore, an increase in the size of the caudate vein has controversial diagnostic value [9].

However, Doppler US is not always sufficient for BCS diagnosis, and further imaging is recommended (contrast-enhanced CT and/or MRI). However, despite a commendable effort to define the background of a diagnostic approach for BCS, currently, there is no universal standard for diagnosis; some recent proposals, referring to catheter venography and surgery, are debatable [10]. Moreover, imaging is not able to rule out small hepatic veins BCS, a common finding in the West, often identified due to an unspecific mosaic enhancement pattern at CT and/or MRI. In many cases, liver histology is the only way to obtain a diagnosis [11,12]. In summary, a pragmatic approach should consider Doppler US as the first diagnostic step, CT or MRI the second, and, finally, liver histology when CT and/or MRI are insufficient [13].

Hepatocellular carcinoma (HCC) is a challenging diagnosis in the context of BCS. However, generally agreed criteria for HCC diagnosis in cirrhosis cannot apply to BCS. In fact, other benign or intermediate lesions, namely regenerative nodules and adenomas, can appear hypervascular in the arterial phase. Consequently, in the context of BCS, histologic confirmation should be indicated in the work-up of HCC [8,9,10,11,12,13,14,15,16,17,18,19,20,21,22]. In future, an effort to find agreeable guidelines for the diagnosis of HCC in BCS should be attempted by physicians and researchers interested in BCS.

## 2. Complications of Budd–Chiari Syndrome

The main complications of BCS are portal hypertension and development of HCC, though the latter is rare [1,2,3,4,5,6,7]. Furthermore, in about 10% of cases, underlying disease could negatively evolve, just like MPD, thus affecting BCS prognosis [23,24]. Additional concerns are bleeding [25] and thrombosis in other organs [24].

## 3. Simplified Clinical Classification and Physiopathology of Budd–Chiari Syndrome

Overall, previous classifications and prognostic indexes of BCS are of limited value, particularly following the widespread implementation of interventional treatments [1,2,3,4,5,6]. A recently proposed pragmatic clinical classification identifies two clinical phases [23]: an asymptomatic (or pauci-symptomatic) phase (AP) or clinically silent thrombosis, and a symptomatic phase (SP). The latter is divided into two stages: a chronic SP (presence of portal hypertension signs but hepatic function preservation) and an acute SP (development of liver failure). Clinically, AP is likely the early phase, as suggested by the early appearance of abdominal and/or subcutaneous portosystemic spontaneous shunts in the absence or signs of symptoms of BCS [5]. In fact, clinical progression is generally a result of subsequent thrombotic extension, although, since BCS severity does not significantly correlate with extension of thrombosis, hepatic functional reserve may also have a significant role [23]. Inflammation is canonically considered the trigger of fibrogenesis in chronic hepatitis. When the main hepatic injury is due to hepatic congestion, without inflammation, parenchymal extinction is proposed as the driver of fibrogenesis [26,27]. Alternatively, fibrogenesis was recently indicated to be driven by chronic hepatic micro-vascular ischemia [28,29,30]. Consequently, liver congestion relief through interventional treatment might be useful as a preventive tool [31,32,33,34].

## 4. Treatments for Budd–Chiari Syndrome

Provided that all the indications of BCS management are derived from retrospective studies [35], following both AASLD and EASL guidelines, BCS is generally supposed to be ruled by a step-by-step management strategy [2,5]. However, whether moving forward in case of no response to therapy is the suggestion, no agreed definition of response to therapy exists [1,2,3,4,5,16,31,32,33], and such a proposal has awaited validation for many years [35].

Anticoagulation is the mainstay of medical therapy, but its efficacy is limited, and it is suitable only for pauci-symptomatic cases. In fact, most cases will eventually require intervention [1,2,3,4,5]. Promising recent data on new oral anticoagulants as treatment of BCS and other splanchnic vein thrombosis also need confirmation [36]. Angioplasty/stenting can be effective only for short-length stenosis [37,38,39,40]. However, TIPS is the main intervention for BCS TIPS [1,2,3,4,5], as stated in early experiences [41,42,43], in published cases with thrombosis extension to the portal vein tree [44,45], in a multi-center European experience on 147 BCS patients [46] and in recently published wide single-center studies [47,48].

Technically, TIPS is an intervention that consists of performing, through a trans-jugular access, a shunt between a hepatic vein and a portal vein branch. Initially, this procedure was applied to relief severe consequence of portal hypertension in cirrhosis. In BCS, due to thrombosis extension, TIPS is usually performed through the caval stump. However, its application to BCS has both theoretical and practical advantages. It has symptomatic and therapeutic potential since, particularly in early stages, it can revert liver congestion, the cause of disease progression in BCS. As most the studies suggest, this also allow TIPS to be effective in BCS with severe liver failure, although TIPS would be generally contraindicated in the setting of cirrhosis [41,42,43,44,45,46,47,48]. However, there are still some are concerns about TIPS for BCS. In fact, as in cirrhosis, TIPS could trigger a further deterioration of liver failure, mostly in late-stage BCS. Furthermore, bleeding due to the procedure is a concrete risk, emphasized by both liver congestion and anticoagulant therapy. However, in selected cases, the advantages of TIPS outweigh the potential risks [41,43,46].

Albeit not generally included in guidelines, traditional surgery has a role in BCS management [49,50,51,52,53,54,55]. In fact, some surgical experience reported fair outcome after surgery, using side-to-side portocaval shunt (SSPCS) [51]. However, SSPCS is not useful for IVC thrombosis, for which SSPCS + cavoatrial shunt, as described in 18 patients (100% long-term survival) or the replacement of the obstructed segment of the IVC with a caval homograft, are the preferred approaches [51,52,53]. Recently, an open surgical reconstruction through exposure of the retrohepatic IVC was used by a single center, reporting commendable results (success in 80/83: 1-, 3-, and 5-year survival rates of 91%, 90%, and 87%) [54].

LT is the last-chance treatment for BCS, once all previous steps have failed, in cases of fulminant liver failure or HCC [55,56,57,58,59,60,61,62,63,64], and published data reports 10-year survival approximating 70% of cases in Europe [60]. Finally, promising, albeit scanty data have been published about living donor LT for BCS [65,66,67].

## 5. Timing of Treatment for BCS

The definition for response to therapy is a critical issue and was not stated by AASLD and EASL guidelines [1,2,3,4,5,23]. In fact, a potential definition based on arbitrary clinical criteria [35] has been awaiting validation for many years. This proposal is based on some clinical arbitrary variables, historically suggested by the Paris group, consisting of the presence of ascites, Na, creatinine, Factor V, bilirubin, portal hypertension bleeding, bacterial peritonitis, and BMI—the so-called Clichy criteria.

However, management and the consequent outcome of BCS are due to these criteria and, according to both a European multi-center published experience and systematic review, about 2/3 of the cases receiving only medical therapy are unlikely to survive long term follow-up [15,68,69].

Furthermore, early intervention, in the presence of clinical portal hypertension, represents a valid alternative [23]. In fact, early TIPS recently had commendable results both in China (100 cases with BCS with diffuse occlusion of HVs) [70] and in the West [71].

With regard to the ongoing debate on the timing of BCS treatment, the sustainers of the step-wise management would argue that using such criteria, TIPS for all would be not needed in 30 to 40% of patients who manage well, long term, without it (anticoagulation with or without angioplasty). Hence, they consider the basis for stepwise procedure as a true personalization based on response to treatment. Moreover, postponing TIPS would not impact survival [24] and the negative impact of massive portosystemic shunting could further deportalize the liver in the context of exuberant regeneration and hepatocellular carcinoma. However, while the application of Clichy criteria was associated with good long-term survival in many centers, there are legitimate doubts about the validity of such criteria for responses which have not been assessed with survival as an endpoint [2,5,24,35].

To resolve the controversies about timing of BCS treatment, two avenues of research could be taken. The former, and theoretically the simplest, is to directly compare prospectively early interventional treatment versus a step-by-step strategy. However, this trial is unlikely to be performed because it should also involve centers where the step-by-step strategy has been adopted for many years with the aim of questioning the stepwise strategy itself. Moreover, the differences of BCS in the West and in the East represent another limitation.

An alternative approach could be to explore which subgroups of patients on only medical therapy would benefit from early intervention (using non-invasive tools capable of addressing the efficacy of intervention for BCS [72,73,74]). To this end, as shown in preliminary findings on four cases, which applied an approach that incorporates elastography, preliminary data would suggest that LS variations from baseline could predict outcome for BCS on sole medical therapy [75]. Further studies could explore whether computational models are useful in the field of BCS [76].
